# Supplementary figures and images for: Ras/ERK and PI3K/AKT signaling differentially regulate oncogenic ERG mediated transcription in prostate cells
Source: PLoS Genet. 2021 Jul 27;17(7):e1009708. doi: 10.1371/journal.pgen.1009708 (PMC8345871; doi:10.1371/journal.pgen.1009708)

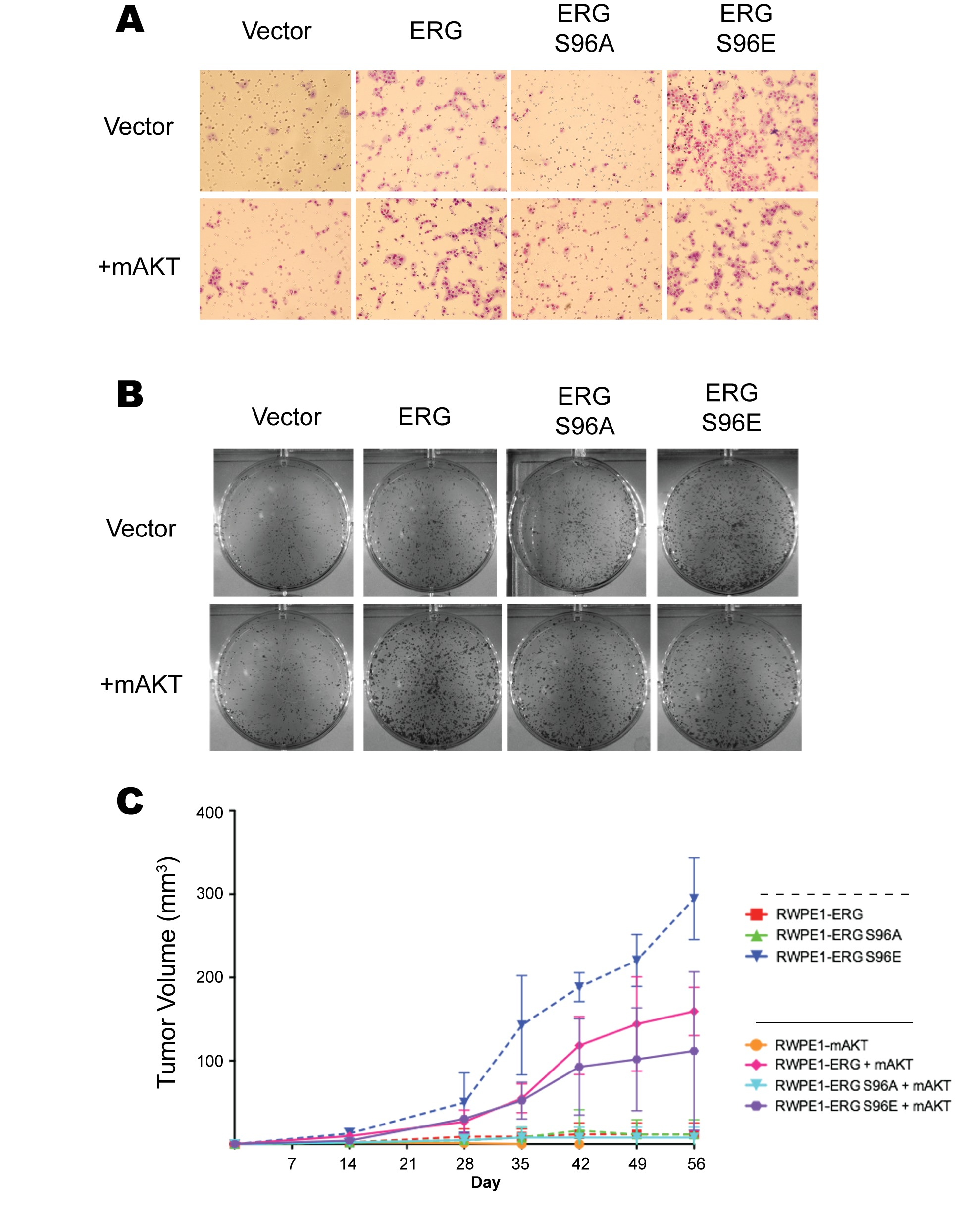

Supplement: S1 Fig — (A) Representative images of migration assays quantified in Fig 1B. (B) Representative images of clonogenic growth quantified in Fig 1C. (C) Weekly xenograft tumor volume measurements of RWPE1 and DLP derived INK4a null cells injected into the flanks of nude mice. (TIF) [file pgen.1009708.s001.tif]

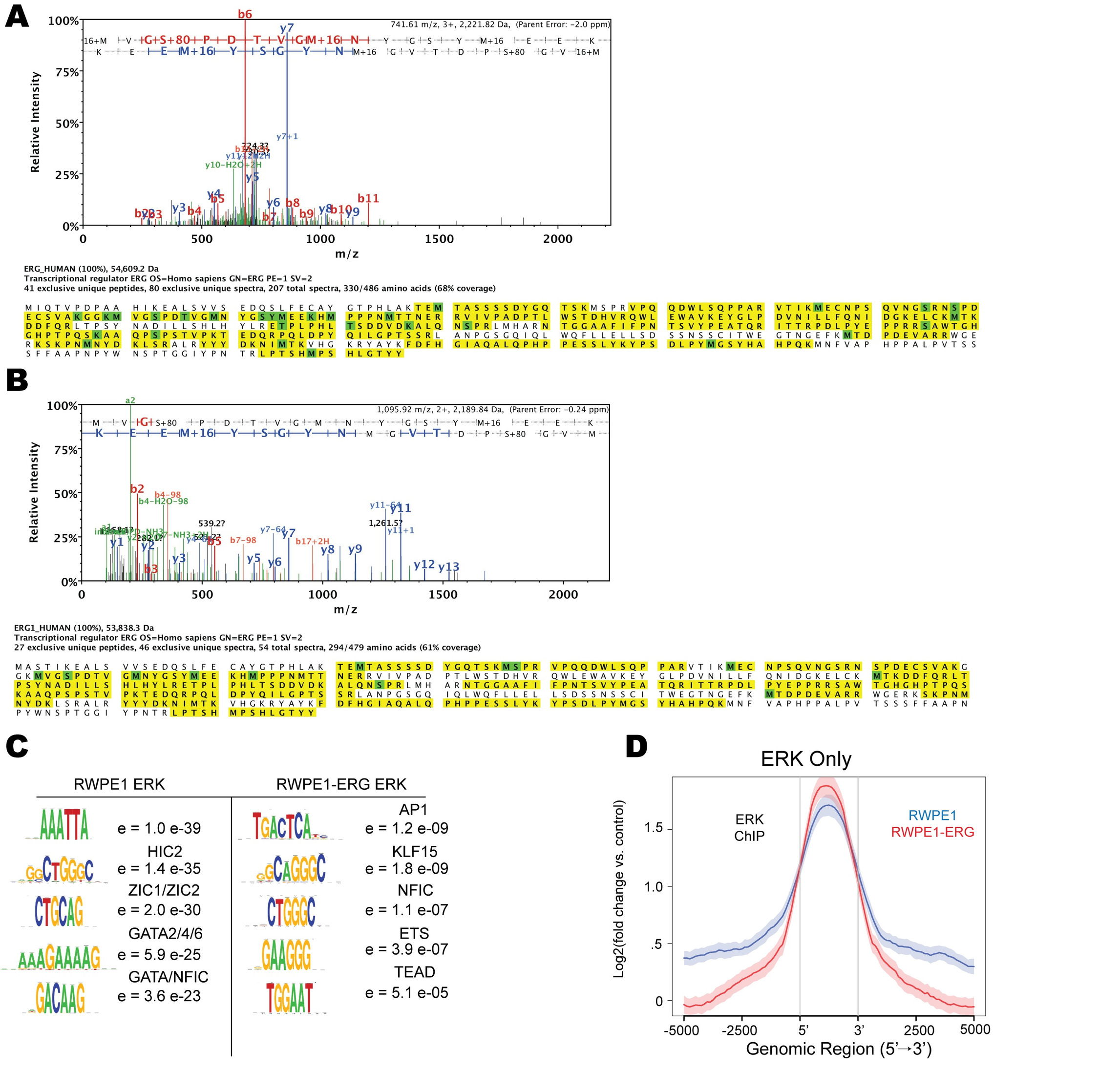

Supplement: S2 Fig — (A) Mass Spectrum identifying phosphorylation of ERG S96 in VCaP and peptide coverage of ERG protein with PTMS highlighted in green. (B) Mass Spectrum identifying phosphorylation of ERG S96 in RWPE1 cells and peptide coverage of ERG protein. (C) Motif analysis of ERK binding sites in RWPE1 and RWPE1-ERG cells. (D) ERK ChIP-Seq coverage centered on regions bound by ERK in both datasets, and not bound by ERG. (TIF) [file pgen.1009708.s002.tif]

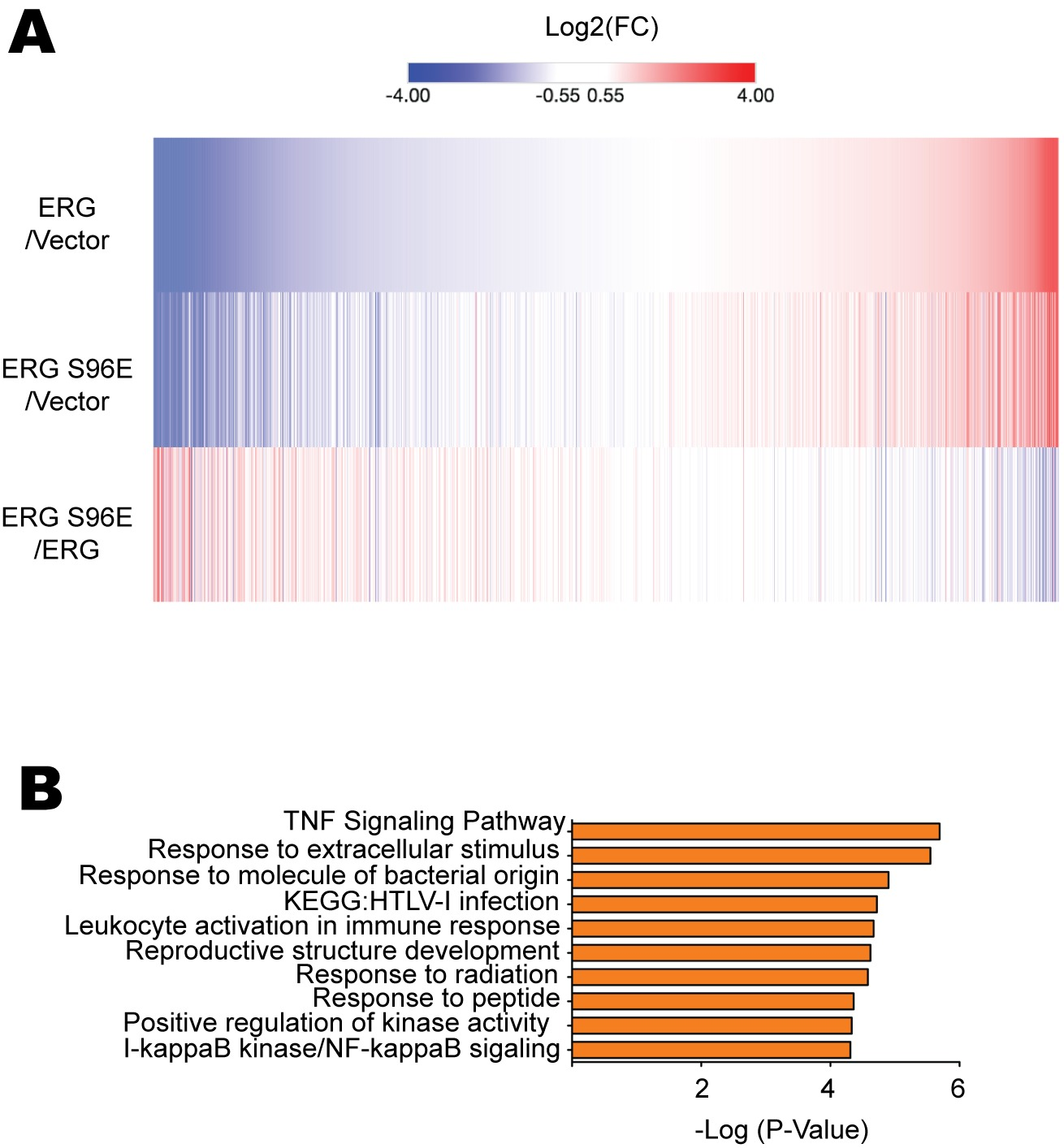

Supplement: S3 Fig — (A) Heatmap of genes nearest ERG bound regions in RWPE1 cells comparing RNA-Seq Log2(FC) of gene expression between RWPE1, RWPE1-ERG, and RWPE1-ERG S96E. (B) Gene Ontology analysis of nearest genes to ERG bound regions in RWPE1 cells that are significantly activated in RWPE1 ERG S96E compared to RWPE1 ERG. (TIF) [file pgen.1009708.s003.tif]

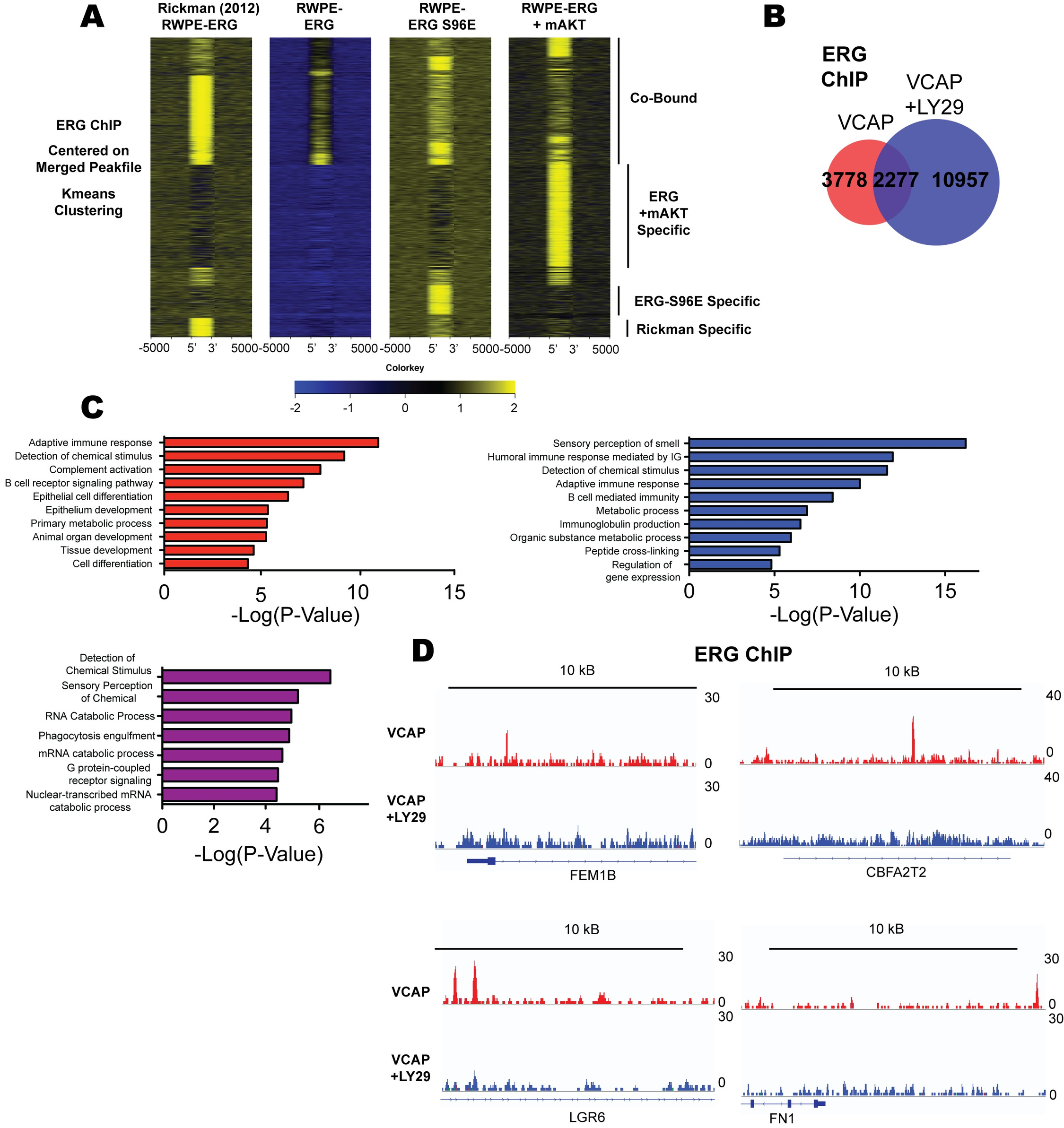

Supplement: S4 Fig — (A) Kmeans clustered heatmap of ERG ChIP-Seq data from indicated lines centered on a combined peak list merged from all four datasets. (B) ChIP-Seq called peaks of ERG in VCaP cells treated with 1uM LY294002 or vehicle for one week and (C) gene ontologies of closest ERG bound gene; ERG co-bound (purple), vehile treated unique (red), and ERG LY294002 treated unique (blue). (D) Gene tracks of ERG ChIP-Seq in VCaP cells treated with vehile (red) or LY294002 (blue). (TIF) [file pgen.1009708.s004.tif]

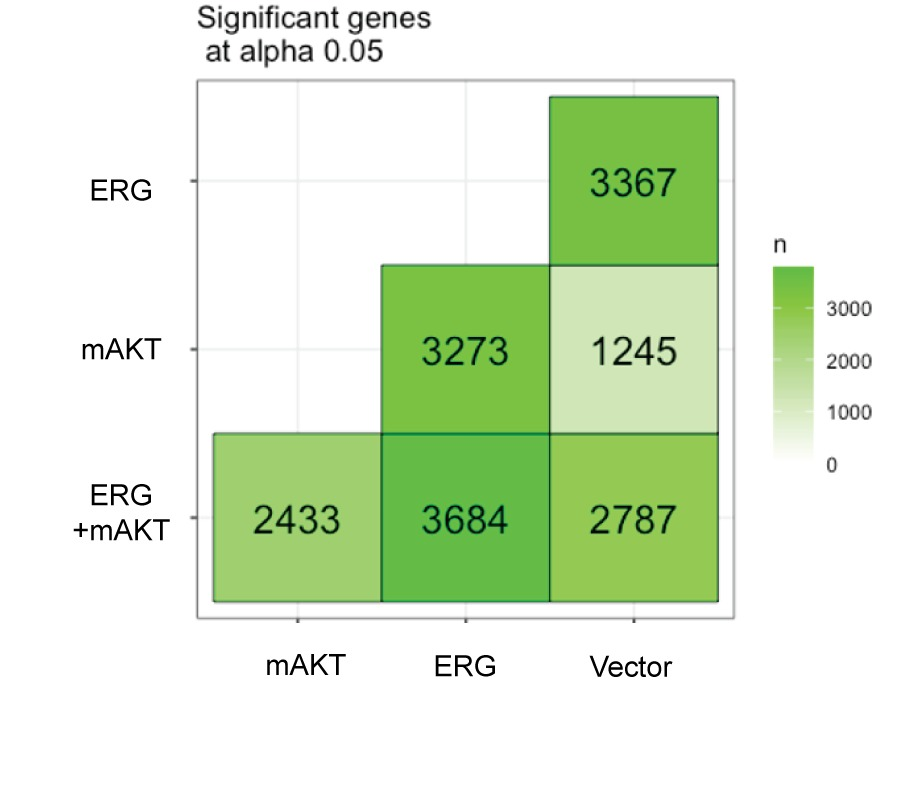

Supplement: S5 Fig — Heatmap Comparison of RNA-Seq data depicting significantly differentially regulated genes between the RWPE1 cell lines indicated. (TIF) [file pgen.1009708.s005.tif]
