# Supplementary material for: Ras/ERK and PI3K/AKT signaling differentially regulate oncogenic ERG mediated transcription in prostate cells
Source: PLoS Genet. 2021 Jul 27;17(7):e1009708. doi: 10.1371/journal.pgen.1009708 (PMC8345871; doi:10.1371/journal.pgen.1009708)
Supplement: Attachment — Submitted filename: Binder1.pdf [file pgen.1009708.s006.pdf]

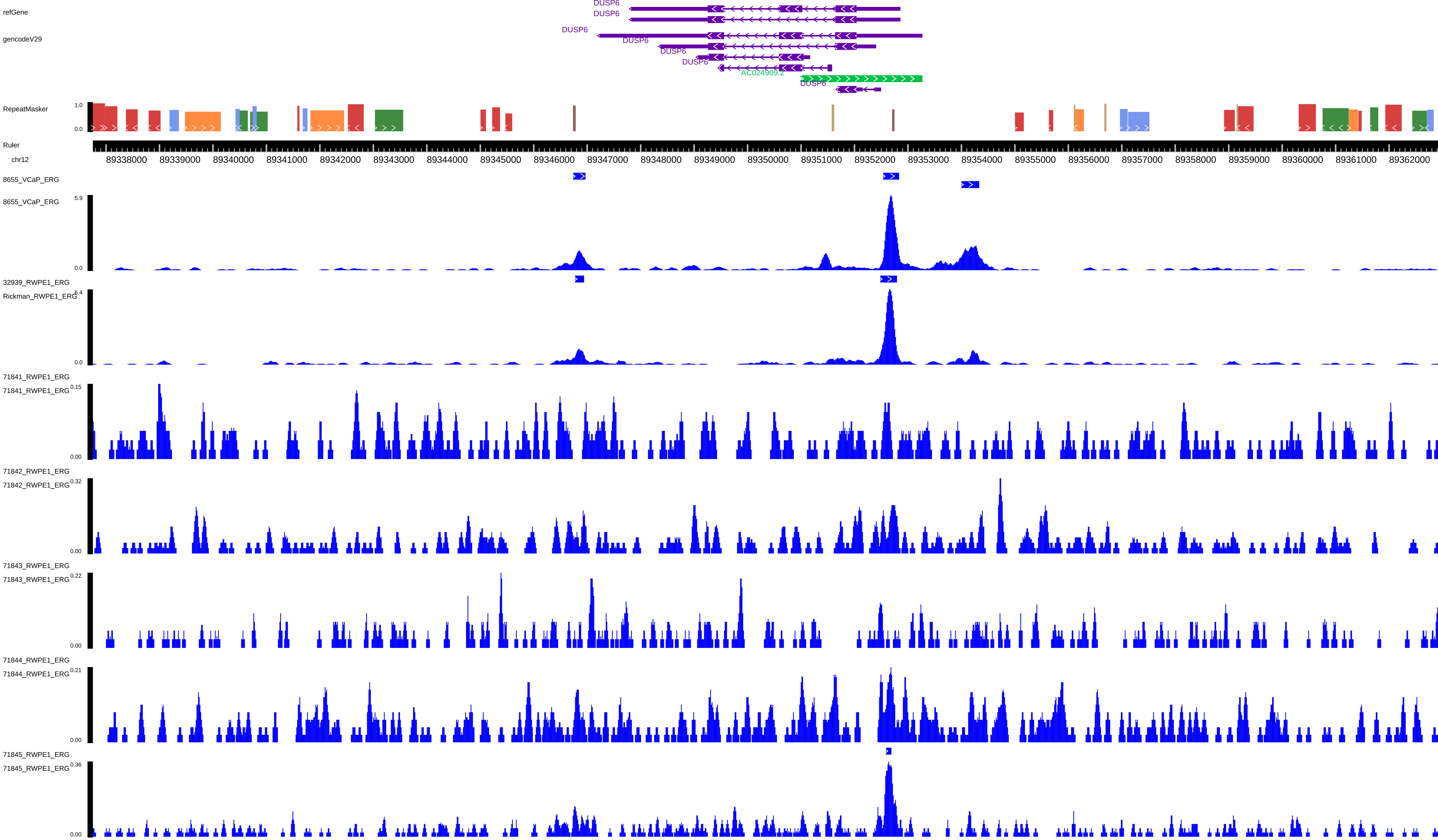

refGene
gencodeV29
DUSP6
AC024909.2
RepeatMasker
1.0
0.0
Ruler
chr12
89338000
89339000
89340000
89341000
89342000
89343000
89344000
89345000
89346000
89347000
89348000
89349000
89350000
89351000
89352000
89353000
89354000
89355000
89356000
89357000
89358000
89359000
89360000
89361000
89362000
8655_VCaP_ERG
5.9
0.0
32939_RWPE1_ERG
Rickman_RWPE1_ERG
6.4
71841_RWPE1_ERG
0.15
0.00
71842_RWPE1_ERG
0.32
71843_RWPE1_ERG
0.22
71844_RWPE1_ERG
0.21
71845_RWPE1_ERG
0.36

Inspector

Title:
Treatment

- ERG-CHIP_RWPE1-ERG(WT)_REP2

ID:
GEO or ENCODE: GSM2195106
CistromeDB: 71844

Species:
Homo sapiens

Citation:
Kedage V, et al. An Interaction with Ewing's Sarcoma Breakpoint Protein EWS Defines a Specific Oncogenic Mechanism of ETS Factors Rearranged in Prostate Cancer. Cell Rep 2016
PMID: 27783944

Factor:
ERG

Biological Source:
Cell Line: RWPE1
Cell Type: Epithelium
Tissue: Prostate
Disease: Normal

Meta-data Correction

Quality Control

Visualize

WashU Browser

UCSC Browser

Download

BED Peaks▾

BIGWIG File▾

Putative Targets

Tools

QC reports

QC motifs

Get top putative targets

Check a putative target

QC	Sample
Raw sequence median quality score	35
% Reads uniquely mapped	86.7%
PCR bottleneck coefficient (PBC)	99.5%
Number of merged Total/Fold 10/Fold 20 peaks	403 / 185 / 46
Fraction of reads in peaks (FRiP)	0.4%
% Peaks in promoter/exon/intron/intergenic	3.6% / 5.5% / 5.5% / 85.4%

QC	Sample
% Top 5k peaks overlapping with union DHS	36.2%
% Top 5k peaks Phastcon Conservation Profiles	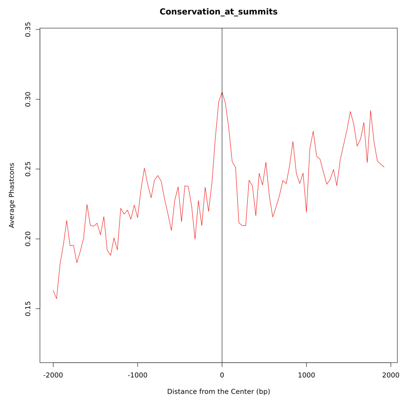

Inspector

Title:
Treatment
- EWS-CHIP_RWPE1-ERG(WT)

ID:
GEO or ENCODE: GSM2195108
CistromeDB: 71843

Species:
Homo sapiens

Citation:
Kedage V, et al. An Interaction with Ewing's Sarcoma Breakpoint Protein EWS Defines a Specific Oncogenic Mechanism of ETS Factors Rearranged in Prostate Cancer. Cell Rep 2016
PMID: 27783944

Factor:
ERG

Biological Source:
Cell Line: RWPE1
Cell Type: Epithelium
Tissue: Prostate
Disease: Normal

Meta-data Correction

Quality Control

Visualize

WashU Browser

UCSC Browser

Download

BED Peaks

BIGWIG File

Putative Targets

Tools

QC reports

QC motifs

Get top putative targets

Check a putative target

QC	Sample
Raw sequence median quality score	39
% Reads uniquely mapped	82.5%
PCR bottleneck coefficient (PBC)	98.4%
Number of merged Total/Fold 10/Fold 20 peaks	391 / 190 / 35
Fraction of reads in peaks (FRiP)	0.4%
% Peaks in promoter/exon/intron/intergenic	0.8% / 0.0% / 4.6% / 94.7%

QC	Sample
% Top 5k peaks overlapping with union DHS	47.3%
% Top 5k peaks Phastcon Conservation Profiles	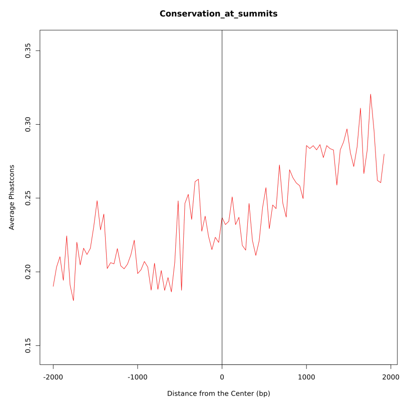

Inspector

Title:
Treatment

- RWPE1-ERG CHIP-SEQ (ERG)

ID:
GEO or ENCODE: GSM927071
CistromeDB: 32939

Species:
Homo sapiens

Citation:
Rickman DS, et al. Oncogene-mediated alterations in chromatin conformation. Proc. Natl. Acad. Sci. U.S.A. 2012
PMID: 22615383

Factor:
ERG

Biological Source:
Cell Line: RWPE1
Cell Type: Epithelium
Tissue: Prostate
Disease: Normal

Meta-data Correction

Quality Control

Visualize

WashU Browser

UCSC Browser

Download

BED Peaks

BIGWIG File

Putative Targets

Tools

QC reports

QC motifs

Get top putative targets

Check a putative target

QC	Sample
Raw sequence median quality score	39
% Reads uniquely mapped	78.2%
PCR bottleneck coefficient (PBC)	95.3%
Number of merged Total/Fold 10/Fold 20 peaks	10036 / 3552 / 367
Fraction of reads in peaks (FRiP)	2.4%
% Peaks in promoter/exon/intron/intergenic	14.0% / 9.3% / 34.4% / 42.2%

QC	Sample
% Top 5k peaks overlapping with union DHS	97.3%
% Top 5k peaks Phastcon Conservation Profiles	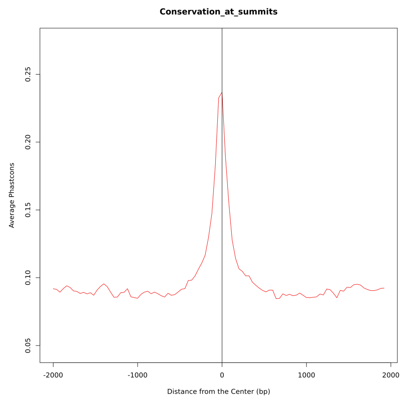
