# Supplementary material for: Ras/ERK and PI3K/AKT signaling differentially regulate oncogenic ERG mediated transcription in prostate cells
Source: PLoS Genet. 2021 Jul 27;17(7):e1009708. doi: 10.1371/journal.pgen.1009708 (PMC8345871; doi:10.1371/journal.pgen.1009708)
Supplement: Attachment — Submitted filename: ResponsetoReviews.pdf [file pgen.1009708.s010.pdf]

Reviewer comment in black, response in red.

Reviewer #2: This revised manuscript is significantly improved and addresses most of my concerns. My only remaining concern rests on the ERG ChIP-seq (Fig 4 and Supplementary Fig 4). This is the basis of expanded ERG cistrome when AKT/PI3K is activated. The use old previously obtained ChIP-seq data from prior publication (ERG and ERG S96E) as a comparator to new ChIP-seq data (ERG + mAKT) done at different time with different cells is not ideal. Ideally, current best practice would call for duplicate ChIP-seq done in parallel. However, another type of analysis that would be satisfactory would be to

1. Use 4 datasets of RWPE-ERG (Kedage 2017), RWPE-ERGS96E (Kedage 2017), RWPE-ERG from Rickman PNAS, and RWPE-ERG-mAKT (this paper).
2. Call peaks. Merge peaks and show merge statistics
3. show peaks of all 4 datasets of teh merged bed file, cluster by k-means to highlight specific peaks in each dataset.

We thank the reviewer for this suggestion, as we think this approach better demonstrates our findings. We have replaced Supplemental Figure S4A with a k-means cluster of all four datasets centered on a merged peak file that combines the peaks from all four datasets. New figure S4A is also shown below for reference. We feel that this analysis shows that the majority of ERG binding signal from the Rickman dataset, our RWPE-ERG, and our RWPE-ERG S96E are common, while there is a large group of new ERG peaks in RWPE-ERG-mAKT cells. We used bedtools MergePeaks to do the merge, and the statistics they provide are also shown below.

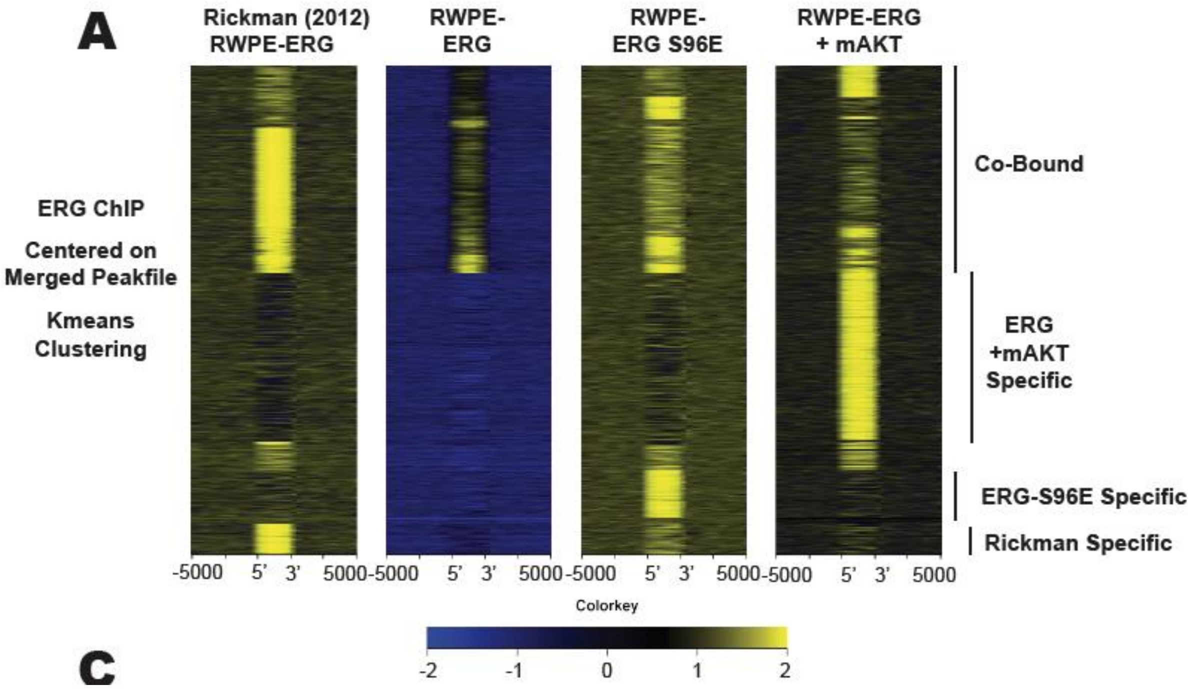

Rickman-ERG.bed	ERG+mAKT.bed	ERG.bed	ERG-S96E.bed	Total	Name
			X	2237	ERG-S96E.bed
		X		387	ERG.bed
		X	X	51	ERG.bed|ERG-S96E.bed
	X			7790	ERG+mAKT.bed
	X		X	102	ERG+mAKT.bed|ERG-S96E.bed
	X	X		12	ERG+mAKT.bed|ERG.bed
	X	X	X	12	ERG+mAKT.bed|ERG.bed|ERG-S96E.bed
X				10769	Rickman-ERG.bed
X			X	729	Rickman-ERG.bed|ERG-S96E.bed
X		X		255	Rickman-ERG.bed|ERG.bed
X		X	X	161	Rickman-ERG.bed|ERG.bed|ERG-S96E.bed
X	X			595	Rickman-ERG.bed|ERG+mAKT.bed
X	X		X	242	Rickman-ERG.bed|ERG+mAKT.bed|ERG-S96E.bed
X	X	X		106	Rickman-ERG.bed|ERG+mAKT.bed|ERG.bed
X	X	X	X	215	Rickman-ERG.bed|ERG+mAKT.bed|ERG.bed|ERG-S96E.bed

[illegible]
