# Supplementary material for: Ras/ERK and PI3K/AKT signaling differentially regulate oncogenic ERG mediated transcription in prostate cells
Source: PLoS Genet. 2021 Jul 27;17(7):e1009708. doi: 10.1371/journal.pgen.1009708 (PMC8345871; doi:10.1371/journal.pgen.1009708)
Supplement: Attachment — Submitted filename: Comments_Weiran Feng.docx [file pgen.1009708.s007.docx]

In this manuscript, the authors aimed at understanding the mechanism of ERG-mediated tumorigenesis in the context of its interaction with Ras/ERK and PI3K/AKT signaling pathways. To this end, they overexpressed ERG (wild-type or ERK1/2-phosphomimetic S96E) with or without an active AKT (mAKT) in RWPE1 cells, a relatively normal human prostate epithelial line. They first documented that ERG-S96E alone and ERG together with mAKT both displays tumorigenic phenotypes. Next, they took chromatin-binding and transcriptomic profiling approaches to reveal that S96E derepressesses ERG targets that are associated with PRC2-mediated silencing without changing ERG chromatin binding, while mAKT creates new ERG chromatin binding and induces luminal fate genes. The observation that ERG S96E promotes tumor formation without a luminal fate change suggests that luminal fates are not necessary for ERG-mediated tumor formation.

Overall this manuscript is well-written and asks important questions in the field, regarding a prevailing prostate cancer oncogene ERG and its roles in tumorigenesis and lineage fate changes. The system used is also well controlled and leads to meaningful interpretations. The study could be improved if the conclusions prove to extend beyond the current one system and translate into patients.

Major comments:

1. All observations are made in the RWPE1 line, raising questions about the generalizability of their conclusions. For example, EZH2 activity is inhibited by ERG expression here (Figure 3), but induced in a different system (Yu et al 2010). It is therefore important to test their conclusions in a different model to evaluate exclude possible artifacts from the current system.
2. The conclusion that PI3K/AKT cooperates ERG to promote luminal fate changes is based on an enrichment of luminal/epithelial genes in ERG/mAKT cells. However, the luminal signature used in Figure 5 is a relative enrichment against mesenchymal cells. This raises the possibility that ERG/mAKT could be actually driving an epithelial fate in general, rather than a bias of a luminal vs basal fate which is more relevant in patients.
3. Related to the above point, the authors also showed AR and FOXA1 expression in ERG/mAKT xenografts. A larger panel of luminal markers and/or tumor histology will be helpful to substantiate their conclusions about luminal differentiation. For example, AR is also expressed in basal and mesenchymal prostate cells so does not serve as a luminal-specific marker.

Minor comments:

1. The figures can be improved if added with more clarity so one does not have to always go to legends or texts for details.
