# Supplementary material for: Ras/ERK and PI3K/AKT signaling differentially regulate oncogenic ERG mediated transcription in prostate cells
Source: PLoS Genet. 2021 Jul 27;17(7):e1009708. doi: 10.1371/journal.pgen.1009708 (PMC8345871; doi:10.1371/journal.pgen.1009708)
Supplement: Attachment — Submitted filename: ResponsetoReviews.docx [file pgen.1009708.s008.docx]

We wish to thank the reviewers and editors for their thoughtful comments. We feel that these comments allowed us to significantly improve the manuscript. Our responses are below in red.

Your manuscript has been reviewed by three expert referees, all of whom find the work of interest, particularly the effects of AKT on the ERG cistrome and induction of luminal gene expression (such as AR and FOXA1). However, several concerns are raised that must be addressed in a revision, specifically:

1) extension of the findings to another model besides RWPE cells, such as VCAP

We have added ERG ChIP-seq in VCaP cells with and without PI3K inhibition. See response to Reviewer #2, comment #2, and Reviewer #3, comment #1.

2) mechanistic insight into how AKT mediates its effects on ERG and induction of luminal gene expression

We have added ATAC-seq in RWPE1, RWPE-ERG, RWPE-mAKT, and RWPE-ERG/mAKT cell lines. See response to Reviewer #2, comment #4.

3) more evidence of luminal lineage specification

We have added additional immunoblots of tumor extracts, and histology. See response to Reviewer #3, comment #3.

In addition, there are significant concerns with the quality of the ERG ChIP data identified by reviewer 2 that must be addressed.

We have added new supplementary Figure 4A, and new files to the GEO database. See also detail in response to Reviewer #2, comment #1.

Reviewer's Responses to Questions

**Comments to the Authors:
Please note here if the review is uploaded as an attachment.**

Reviewer #1: In the present study the authors aimed to assess the impact of ERK and AKT pathways on ERG function in chromatin binding, gene repression/transactivation and tumorigenesis. They found that ERK co-localized with ERG in the nucleus and in a subset of ERG target genes and ERK phosphorylation of ERG at S96 results in loss of ERG association with EZH2 and H3K27me3 repression histone mark. They further showed that S96E is important for ERG mediated tumorigenesis, but this effect is unlikely caused by changes in ERG cistrome. In contrast, AKT expression largely induces ERG cistrome reprogramming and most strikingly, co-expression of AKT with ERG converts AR-null RWPE1 cells into AR-positive luminal cells, although the authors point out that the underlying mechanism is not entirely clear.

Overall, it is a very exciting study and data are quite convincing. The data are well interpreted and the conclusion is supported by the data presented. Only a few minor issues need to be addressed.

We are pleased that the reviewer found the study exciting and the data convincing.

The data shown in Figure 3A is very intriguing. It is important to determine whether S96E phosphomimetic mutant induced decrease in EZH2 binding and H3K27me3 levels at ERG target promoters and enhancers is statistically significant.

We thank the reviewer for this point. We ran T-tests on the H3K27me3 sequencing data enrichment between all 3 conditions at both promoters and enhancers. ERG-S96E expression was found to significantly (p=0.5) decrease H3K27me3 at ERG bound promoters compared to WT-ERG and at enhancers WT-ERG expression resulted in a significant depletion (p<0.1) of H3K27me3 which was not further significantly depleted by ERG-S96E expression. These p-values are now marked in Figure 3A.

It would be more informative if top 5 or 10 genes activated by S96E are marked in Figure 3C.

We have now labeled the 5 most highly differentially expressed genes between ERG-S96E and ERG in Figure 3C.

In Line145 in the phase of “S103 in Uniprot isoform 1”, it should be S103 in isoform 2. Since ERG isoform 2 is the one most frequently detected in TMPRSS2-ERG fusions in patients and the numbering of phosphorylation site is likely based on isoform 1, it is important for authors to indicate in the upfront of the Results section that ERG isoform 1 was used in the current study and briefly mention the differences between ERG isoforms, especially with isoform 2.

We thank the reviewer for identifying this mistake. Yes, we used isoform 1 for our numbering to remain consistent with our previous publications. We now detail in the results section the difference between isoform 1 and 2.

Figure 2B in Line 298 should be Figure 4B.

Thank you for identifying this error, it has been fixed.

In line 333 and 335, it seems the description of the results does not match to the figures cited.

We apologize for this error, these lines now correctly reference Figure 5C.

The paper reported by Blee et al. (Clin Cancer Res 2018) should be cited in the context of discussion of the role of ERG in promoting luminal epithelial phenotype, especially in cells with PTEN loss.

We apologize for this oversight, this paper has now been cited in the discussion regarding the role of ERG in promoting luminal epithelial phenotype.

Reviewer #2: Prostate cancer is characterized by ERG overexpression through translocation in ~ half of all cases. There is co-occurrence with PTEN loss and several model systems have recapitulated cooperativity between PTEN loss and ERG overexpression in tumorigenesis. This manuscript addresses the role of PI3K/AKT as well as Ras/ERK in ERG function.
Overall, the manuscript tells two separate stories. The first is the role of ERG phosphorylation on S96 in tumorigenesis and the second is the role of AKT activation on ERG activation and binding. Much of the data in the first story has been previously published in Kedage et. al. JBC 2017, including that ERG S94E by itself stimulates migration, gene activation, and decreased PRC2 suppression at sites. The second story is more novel and potentially impactful. It is quite interesting that adding of both ERG and myrAKT in RWPE cells causes expression of FOXA1 and AR. However, the data presented is not well flushed out and lacks quality controls.
Major Comments:
1. I am quite worried about the quality of ChIP-seq that has led to the conclusions in the manuscript. ERK ChIP peaks in Fig 1C and 1E are very broad, though that may be it’s true biology. More concerning are ERG peaks in Fig. 4A, which should be sharp but seem very broad. The authors did not make the raw files for reviewers to examine. However, this reviewed did go into the [Cistrome.org](http://Cistrome.org) public repository where ERG ChIP-seq in RWPE cells were deposited from this lab’s prior work as well as from the lab (Kedage et. al., Cell Rep 2016) of David Rickman (Rickman et. al. PNAS 2012). It is worrisome that the deposited samples from Kedage et. al did not meet quality control of [cistrome.org](http://cistrome.org) for # peaks, peaks at known open regions, etc. For authors, I’ve attached a PDF file, 1st page of the DUSP6 locus with known ERG peaks in all lineages. Top two tracks are VCAP and Rickman RWPE and below are tracks from Kedage et. al. The next 3 pages are QC metrics from [cistrome.org](http://cistrome.org) of Rickman RWPE and Kedage RWPE. For resubmission, authors need to submit all the QC metrics of their ChIP-seq as well as bed files for where the peaks are and bigwig files.

We thank the reviewer for helping us verify the rigor of this work. We conducted FastQC of all ChIP files and all samples were found to pass basic QC controls. ERG ChIP-seq from Kedage et al. Cell Reports 2016 was not analyzed or used to make any conclusions in this manuscript. ERG ChIP and ERG-S96E ChIP-seq from Kedage et al. JBC 2017 were analyzed. These sequencing files passed FastQC. In addition, we have now plotted these ERG ChIP data centered on the ERG ChIP from Rickman PNAS 2012 and our samples exhibited similar coverage (Supplemental Figure 4A). Using IGV to view our ERG ChIP we observe a peak at the DUSP6 locus (below). FastQC metrics for sequencing experiments are included below. Although our sequencing experiments did not have as much sequencing depth as Rickman PNAS 2012, our ChIP-Seq experiments each had > 30 million reads. All bed files and bigwig files are in GEO accession number GSE164859 with reviewer access token mpepeoamrjwdvax.

ERG-S96E

ERG

RWPE1-ERG

ERG ChIP

RWPE1-ERG +mAKT

ERG ChIP

RWPE1-ERG S96E

ERG ChIP

RWPE1-ERG

ERK ChIP

RWPE1

ERK ChIP

RWPE1

H3K27me3 ChIP

RWPE1-ERG S96E

H3K27me3 ChIP

RWPE1-ERG

H3K27me3 ChIP

2. Essentially all the work has been done in RWPE cells, and its relevance is questionable. It is well known that VCAP ERG cistrome and transcription is very distinct from RWPE. The findings should be recapitulated in VCAP cells.

We thank the reviewer for this good suggestion. To investigate whether PI3K/AKT signaling altered the ERG cistrome in a cell line with an endogenous *TMPRSS2-ERG* rearrangement, VCaP cells were treated with either DMSO or 1 μM PI3K inhibitor (LY290024) 3 times over the course of a week and then subjected to ERG ChIP-Seq. Overall, we found that PI3K inhibition did not shrink the ERG cistrome but resulted in a substantial differential binding of ERG (New supplemental Figure 4B). ERG peaks unique to DMSO treated VCaP cells were enriched with gene ontologies related to epithelial differentiation and development, whereas ERG peaks unique to LY294002 treated cells did not contain enrichment for epithelial ontologies (New supplemental Figure 4C). We also identified individual ERG peaks at epithelial differentiation genes that were lost upon PI3K inhibition (New supplemental Figure 4D).

3. PTEN loss followed by PIK3CA and PIK3R1 mutations are clinically relevant alterations of the PI3K pathway. Can authors recapitulate at least the phonotype using these alterations?

This is an interesting question, however we were not able to test this within the timeframe of revisions for this manuscript.

4. The increase in ERG binding sites in RWPE expressing mAKT is striking and the most novel and impactful part of the paper. This finding is in contrast to prior mouse models (Chen et. al, Nat Gen). There is no mechanistic insight. Are the new sites a reflection of opening of chromatin and change in the open chromatin landscape by mAKT or increased ERG binding to established enhancer/promoters? In RWPE with mAKT and also in VCAP’s, does ERG cistrome shrink upon inhibition of AKT?

Like the reviewer, we also hypothesized that mAKT would alter the chromatin landscape. To test this, we have now conducted ATAC-Sequencing in RWPE1, RWPE1-ERG, RWPE1+mAKT, and RWPE1-ERG+mAKT to interrogate any such changes. Surprisingly, all four ATAC-sequencing experiments exhibited a high degree of overlap (New Figure 4E). Interrogating only regions newly bound by ERG in the presence of mAKT, we did not identify any evidence of alterations in open chromatin upon the addition of mAKT (New Figure 4E). We also looked at individual ERG binding sites by IGV and did not identify any changes in chromatin accessibility (2 examples shown in new Figure 4F). These data suggest that novel ERG binding is not due to alterations in chromatin accessibility. We hypothesize that these changes may be due to altered interactions with other transcription factors. As mentioned in the response to question 2, in VCaPs, the ERG cistrome changes, but does not shrink upon inhibition of the PI3K/AKT pathway (new supplemental Figure 4B). Because of this, we have modified the language in the abstract to indicate a change in, rather than an expansion of, the ERG cistrome.

5. Specifically, can ERG phosphorylation be inhibited by novel ERK kinase inhibitors and does that change ERG mediated cistrome/transcriptome

In our previous study (Kedage et al. JBC 2017) we found that phospho-null and phospho-mimetic mutations of ERG had no effect on the ERG cistrome, but did alter the ERG mediated transcriptome. In that study we focused only on ERG-activated genes and found that ERG-S96A failed to activate these genes, while ERG-S96E maintained activation. We would predict that utilizing a ERK kinase inhibitor would have a similar effect as ERG-S96A to the ERG cistrome/transcriptome.

6. In fig 1D-E, In vivo tumor formation was done with RWPE cells and immortalized MEFs or CAFs. Authors need to show histology to confirm that tumors are epithelial and not sarcomas from the fibroblasts.

We thank the reviewer for this suggestion. We now show histology in new Figure 6D. We stained for pan-cytokeratin, which shows the tumors are epithelial. We do see some Vimentin staining in the RWPE-ERG S96E tumors, which could be from the stromal cells.

7. In Fig 2E, authors need to show ERK phosphorylation is not changed by ERG expression (ERG can induce feedback genes that inhibit ERG phosphorylation) and that ERK ChIP-seq signal at non-ERG sites is unchanged (i.e., change is specific to ERG sites).

We measured ERK phosphoryation in a previous publication using these same cell lines (Selvaraj et al. Molecular Cancer 2014). We did observe a small induction of ERK phosphorylation upon ERG expression (Selvaraj et al Figure 1). As the reviewer mentions, this is consistent with a positive feedback loop that we describe in detail in a more recent publication (Greulich et al. NAR Cancer 2021). We do not think that this change in phosphorylation alters any of the conclusions in this manuscript. To address the second part of this comment, we plotted the average ERK ChIP-seq signal at sites bound by ERK, but not ERG (new supplemental Figure 2D), using the same methodology as the plot in Figure2E. We do not observe the increase in enrichment of ERK in the cell line expressing ERG at these non-ERG sites.

8. Fig 5A is hard to interpret for overall gene expression.. A unsupervised clustering and correlation clustering should also be shown.

We thank the reviewer for this suggestion. Unsupervised clustering was conducted of the RNA-Seq data from our RWPE1 cell lines and revealed that RWPE1-Vector and RWPE1-ERG clustered together whereas RWPE1+mAKT and RWPE1-ERG+mAKT formed a separate cluster. This is shown in new Figure 5A, and the old Figure 5A is now Supplemental Figure 5.

9. (Jerde et al. 2009) not in the reference list

We apologize for this error. This publication has been added to the reference list.

Reviewer #3: In this manuscript, the authors aimed at understanding the mechanism of ERG-mediated tumorigenesis in the context of its interaction with Ras/ERK and PI3K/AKT signaling pathways. To this end, they overexpressed ERG (wild-type or ERK1/2-phosphomimetic S96E) with or without an active AKT (mAKT) in RWPE1 cells, a relatively normal human prostate epithelial line. They first documented that ERG-S96E alone and ERG together with mAKT both displays tumorigenic phenotypes. Next, they took chromatin-binding and transcriptomic profiling approaches to reveal that S96E derepressesses ERG targets that are associated with PRC2-mediated silencing without changing ERG chromatin binding, while mAKT creates new ERG chromatin binding and induces luminal fate genes. The observation that ERG S96E promotes tumor formation without a luminal fate change suggests that luminal fates are not necessary for ERG-mediated tumor formation.

Overall this manuscript is well-written and asks important questions in the field, regarding a prevailing prostate cancer oncogene ERG and its roles in tumorigenesis and lineage fate changes. The system used is also well controlled and leads to meaningful interpretations. The study could be improved if the conclusions prove to extend beyond the current one system and translate into patients.

Major comments:

1. All observations are made in the RWPE1 line, raising questions about the generalizability of their conclusions. For example, EZH2 activity is inhibited by ERG expression here (Figure 3), but induced in a different system (Yu et al 2010). It is therefore important to test their conclusions in a different model to evaluate exclude possible artifacts from the current system.

We thank the reviewer for this suggestion. As in the response to reviewer 2, question 2, we now test the role of the PI3K/AKT pathway in ERG binding in the VCaP cell line model (new supplemental Figure 4B, C, and D). We also compare RWPE1 to VCaP in Figure 2A. We would also like to point out that in our previous paper (Kedage et al. 2017), we observed no change in EZH2 occupancy at non-ERG targets when we introduced ERG or ERG mutants. Our findings are consistent with a possible increase in EZH2 function at non-ERG targets and do not conflict with previous reports of an oncogenic role for EZH2.

2. The conclusion that PI3K/AKT cooperates ERG to promote luminal fate changes is based on an enrichment of luminal/epithelial genes in ERG/mAKT cells. However, the luminal signature used in Figure 5 is a relative enrichment against mesenchymal cells. This raises the possibility that ERG/mAKT could be actually driving an epithelial fate in general, rather than a bias of a luminal vs basal fate which is more relevant in patients.

We thank the reviewer for indicating that we need to clarify this point. Yes, in Figure 5 (and elsewhere) we show that the addition of ERG to RWPE1 makes the cells more mesenchymal, and the addition of AKT to ERG (RWPE ERG/mAKT) decreases EMT and increases epithelial and luminal epithelial markers in relation to this. However, Figure 5C also shows that the addition of AKT alone to RWPE1 decreases EMT and increases "epidermal" signatures. The addition of ERG to AKT (far right panels) increases the luminal signature in relation to RWPE mAKT - i.e. in cells that were already more epithelial than RWPE1.

3. Related to the above point, the authors also showed AR and FOXA1 expression in ERG/mAKT xenografts. A larger panel of luminal markers and/or tumor histology will be helpful to substantiate their conclusions about luminal differentiation. For example, AR is also expressed in basal and mesenchymal prostate cells so does not serve as a luminal-specific marker.

We now include tumor histology (new Figure 6D) and immunoblot of the basal marker TP63 (Figure 6C). TP63 expression is very interesting, it is high in tumors expressing ERG S96E and absent in tumors expressing ERG S96A, consistent with a direct role of ERG in repression of TP63, as reported in Li et al. JCI 2020. The histology shows very different morphology between ERG S96E tumors and ERG/mAKT tumors. Importantly, we observe high expression of the basal cytokeratin 5 (KRT5) in the ERG S96E tumors, and much lower expression in the ERG/mAKT tumors. Unfortunately, we could not observe the luminal cytokeratin KRT8, despite a previous report that it is expressed in RWPE1 cells (Bello et al. 1997). We are not sure if this is because KRT8 is not expressed in the tumors, or if our staining failed. We used mouse prostate glands as a positive control, but it is possible the antibody stains mouse KRT8 better than human KRT8. Because of this, we blunted the language in this section to say that ERG/mAKT results in loss of basal characteristics and gain of androgen receptor expression, rather than gain of luminal cell fate. Our RNA-seq data from the cell lines still show gain of a luminal gene expression program.

Minor comments:

1. The figures can be improved if added with more clarity so one does not have to always go to legends or texts for details.

We thank the reviewer for this suggestion. We have added labels to many of the figures to make the results more clear without consulting the legends or text.
